# Glaucoma in a Suburban Tertiary Care Hospital in Nigeria

**Published:** 2010-04

**Authors:** Malachi Epo Enock, Afekhide Ernest Omoti, Razak Onozutu Momoh

**Affiliations:** 1 Irrua Specialist Teaching Hospital, Irrua, Edo State, Nigeria; 2 University of Benin Teaching Hospital, Benin City, Edo State, Nigeria

**Keywords:** Glaucoma, Morbidity, Types, Irrua

## Abstract

**Purpose:**

To determine the incidence and contribution of different types of glaucoma to blindness at Irrua Specialist Teaching Hospital, a suburban tertiary care hospital in Edo State, Nigeria.

**Methods:**

Medical records of all new patients with glaucoma who presented to the eye clinic of the hospital from June 2007 to May 2009 were reviewed.

**Results:**

Out of a total of 2,742 new patients seen over the study period, 177 (6.5%) subjects had glaucoma which included primary open angle glaucoma (130 cases, 73.4%), juvenile glaucoma (31 patients, 17.5%), secondary glaucoma (10 subjects, 5.6%), congenital glaucoma (3 cases, 1.7%) and primary angle closure glaucoma (3 persons, 1.7%). Of patients with primary open angle glaucoma, 23 (17.7%) were blind based on visual acuity criteria and 67 (51.5%) were blind based on visual field criteria.

**Conclusion:**

Glaucoma remains a blinding scourge; late presentation, especially in rural areas, is an important factor predisposing to blindness. In this Nigerian population primary open angle glaucoma was the most prevalent subtype of the disease but primary angle closure glaucoma was rare.

## INTRODUCTION

The glaucomas are classified as open-angle or angle-closure depending on the mechanism by which aqueous outflow is impaired, and as primary or secondary depending on the presence or absence of associated factors affecting aqueous outflow. Furthermore, age at onset may be used to classify glaucoma into congenital, infantile, juvenile or adult forms.^1^

The World Health Organization estimates that 105 million people are glaucoma suspects, about 13.5 million people over the age of 40 years have primary open angle glaucoma (POAG) which constitutes 60% of the total burden of the disease, 6 million (26.6%) people have primary angle closure glaucoma (PACG), 300,000 children (1.3%) have congenital glaucoma, and 2.7 million individuals (12.1%) are affected with secondary glaucomas. Globally, approximately 70% of POAG cases belong to developing countries.^2^ The number of people with primary glaucoma in the year 2000 was estimated to be 66.8 million with 6.7 million subjects suffering from bilateral blindness.^3^

Glaucoma is the second leading cause of visual loss and blindness in the world following cataracts.^4^ Previous studies in Nigeria using visual acuity criteria showed that glaucoma is responsible for 17.1–43.3% of bilateral blindness.^5^–^8^

This clinic based study was carried out at the Irrua Specialist Teaching Hospital, Irrua, Edo State, Nigeria. This suburban tertiary care hospital serves as the only referral center for the Edo North and Central senatorial zones, and parts of Ondo, Koji and Delta States in Nigeria. This retrospective study aims to determine the incidence of different types of glaucoma in Irrua and their contribution to visual loss.

## METHODS

Medical records of all new referrals to the eye clinic of Irrua Specialist Teaching Hospital, Edo State, Nigeria, from June 2007 to May 2009 were reviewed. The records of subjects diagnosed with glaucoma were retrieved and analysed for the type of glaucoma. All cases included in the study had gonioscopic examination using a Goldmann-type goniolens, either at presentation or subsequently.

Diagnostic criteria for POAG included glaucomatous optic nerve damage (cupping and atrophy), visual field loss, normal appearance of the anterior chamber angle on gonioscopy, adult onset and absence of secondary causes of open angle glaucoma. PACG was diagnosed in an eye with an occludable drainage angle and features indicating trabecular obstruction by the peripheral iris, such as peripheral anterior synechiae, elevated intraocular pressure (IOP), iris whorling (distortion of radially orientated iris fibers), “glaucomfleken” lens opacities, or excessive pigment deposition on the trabecular meshwork together with evidence of glaucomatous optic nerve damage and visual field loss.^9^ Secondary glaucoma was diagnosed when there was raised IOP in the presence of an obvious cause. Congenital glaucoma was diagnosed in the presence of raised IOP and buphthalmos. IOP in congenital glaucoma was assessed digitally in the first instance and then by the Reichert Tono-pen XL applanation tonometer (Reichert Inc., Depew, New York, USA) under general anesthesia. Juvenile glaucoma was diagnosed based on similar findings as POAG, but age of onset in children and young adults (between 2 and 30 years of age) for the purpose of this study. Fortunately, all patients with juvenile glaucoma were at least 10 years of age and hence readily complied for gonioscopy, visual field evaluation and IOP measurement.

Visual acuity was measured using a Snellen chart; threshold perimetry was obtained with the Synemed EP- 910 automated visual field analyzer (Synemed Inc., Benicia, California, USA) and interpretation was performed by examining the pattern deviation. In very advanced cases, visual fields were assessed by confrontation. IOP was measured using the Goldmann applanation tonometer mounted on a slitlamp and the Reichert Tono-pen XL applanation tonometer in subjects with congenital glaucoma. Gonioscopy was done using a Goldmann 3-mirror contact lens in older children and adults, and fundoscopy was performed either with the Keeler specialist (Keeler LTD, Windsor, UK) or the Welch-Allyn (Welch-Allyn Inc., Skaneateles Falls, New York, USA) ophthalmoscopes. Glaucomatous optic disc atrophy was confirmed by stereoscopic examination of the optic disc with a +90 diopter lens at the slitlamp.

Information on age, sex, cause of secondary glaucoma, visual field defects and IOP at presentation were recorded. Glaucomatous optic disc appearance was graded using the modified grading system proposed by Jay^10^ as follows: stage 1, suspicious shape of the optic cup but cup/disc ratio less than or equal to 0.6; stage 2, pathological cup/disc ratio greater than 0.6 but less than 0.9; and stage 3, end-stage cupping with cup/disc ratio greater than or equal to 0.9.

An arbitrary classification of visual field damage modified by Jay^10^ was also used as follows: stage 1, minimum field loss consisting of early arcuate defects or nasal steps; stage 2, defects greater than 10° from fixation; stage 3, visual field constriction to less than 10° from fixation; stage 4, defects resulting in visual field constriction to less than 5° from fixation in 1–3 quadrants; and stage 5 defects resulting in visual field constriction to less than 5° in all quadrants including subjects who cannot fixate on the target and are found to have grossly constricted fields by confrontation. Stages 3 to 5 were classified as blind by visual field criteria.

## RESULTS

A total of 2,742 patients including 1,470 (53.6%) male and 1,272 (46.4%) female subjects were referred over the 2-year period of the study. Of these, 177 (6.5%) patients had glaucoma including 107 (60.5%) male and 70 (39.5%) female subjects. Twenty-two patients with glaucoma were excluded because gonioscopy was either not performed or the information provided was inadequate to classify them into the various subgroups.

As shown in Table 1, POAG was the most common type of glaucoma (73.4%), followed by juvenile onset open angle glaucoma (17.5%). Age and sex distribution of all patients with open angle glaucoma is shown in Table 2. The peak incidence was seen in the sixth decade of life. Twenty-three patients (17.7%) with adult onset POAG were bilaterally blind at presentation based on visual acuity criteria (no light perception to less than counting fingers at 3 meters). Cup/disc ratio was ≤0.6 in 51 (39.2%) patients, 0.7–0.9 in 42 (32.3%) patients and ≥0.9 in 37 (28.5%) patients. Table 3 summarizes the stage of glaucomatous defects based on visual fields in the better eye. Thirty-five (26.9%) patients could not fixate on the target due to very poor vision, implying that 67 (51.5%) patients had bilateral blindness based on visual field criteria.

Mean IOP in subjects with glaucoma was 31.1±12.3 (range, 12–57) mmHg; IOP was less than 21 mmHg in the eye with higher IOP in 35 (22.6%) patients.

PACG was seen in 3 (1.7%) patients. All of them were female and presented with typical features of angle closure glaucoma. Three (1.7%) patients had congenital glaucoma including 2 female infants 2 and 5 months of age, and one male infant 3 months of age at presentation. All of them had bilateral buphthalmos and hazy corneas with corneal diameters of 13 mm, 12.5 mm and 13.4 mm, respectively. IOP measurement under anesthesia revealed pressures higher than 28 mmHg in these children.

Secondary glaucomas were present in 10 (5.6%) patients including 6 male and 4 female subjects. Traumatic hyphema, couching and traumatic iritis were responsible for two cases each; traumatic anterior lens dislocation, intumescent cataract, neovascular glaucoma and complicated cataract surgery were responsible for one case each (Table 4). The complicated case of cataract surgery was due to anterior uveitis. All cases of secondary glaucoma, with the exception of the patient with neovascular glaucoma, were unilateral. In this category, four patients (all male with mean age of 35.3 years) had monocular blindness.

## DISCUSSION

In the current study, POAG was the most common form of glaucoma. Previous studies elsewhere in sub-Saharan Africa have shown that it accounts for at least 45% of all cases of glaucoma.^11^–^15^ We observed that in the outpatient eye clinic of the Irrua Specialist Teaching Hospital, it accounted for 73.4% of all cases. This value is similar to the 76.9% recently reported from Benin-City,^16^ but higher than the 55.6% rate reported in Lagos^17^ some decades ago. This may be not only due to better facilities now available for early diagnosis of the disorder, but also due to the positive impact of greater public awareness of the disease causing patients to present earlier for screening. Furthermore, the positive impact of frequent free eye camps organized by different non-governmental organizations, especially in rural areas, cannot be over-emphasized. Glaucoma suspects are usually referred to our eye clinic from such eye camps. The findings of this study are therefore in line with global data on glaucoma placing POAG as the most common type.^2^

The peak incidence of glaucoma was in the sixth decade of life. This is also in agreement with findings in Benin-City^16^ and Lagos.^17^ About one-third of the patients presented before the age of 40 years. These findings agree with previous studies showing that the onset of POAG in Africans is 1 to 2 decades earlier than Caucasians.^16^,^17^ At presentation, 17.7% of our patients were blind in both eyes based on visual acuity criteria. This is similar to the 16.8% figure in a recent study from Benin-city,^16^ but less than the 34% rate reported in Kaduna^18^ about 3 decades ago and the 40.6% rate reported in Ibadan^19^ almost 4 decades ago. Apart from improved awareness and level of education, the increased availability of ophthalmology services, even in rural and suburban areas is an important factor. Also, different visual acuity definitions were employed for blindness; while the Kaduna study used visual acuity of counting fingers at less than 1 meter to define blindness, the Ibadan study employed visual acuity of less than counting fingers at 3 feet, which was similar to our criteria. Using visual field criteria, 51.5% of patients were blind in both eyes upon presentation in our series which is similar to the recent study in Benin-City^16^ in which 41.6% of subjects were blind and the study in Onitsha,^20^ Eastern Nigeria, just over a decade ago in which 44.2% of patients were blind at presentation. This underscores the fact that earlier studies which defined blindness only using visual acuity criteria grossly underestimated the visual disability resulting from glaucoma.

PACG occurred only in 3 (1.7%) patients in this study. This is similar to the findings in Benin-City,^16^ Lagos^17^ and other parts of Africa^11^–^14^,^21^ supporting earlier reports indicating that PACG is rare in Africans.^22^ This rarity still remains unexplained. It is also possible that if all the patients had complete information and were not excluded, more patients with PACG, especially of the chronic variety, may have been identified. In older studies, PACG was not defined by optic nerve damage which is in contrast to the current definition.^1^,^9^ This inconsistency may account for the higher incidence of PACG in some older studies and can be a source of confusion when comparing recent reports with previous ones.^1^

In our series, 3 (1.7%) patients had congenital glaucoma and all of them had bilateral buphthalmos at presentation. Most of our patients tend to present with advanced disease, poor visual acuity and buphthalmos.^16^,^17^ Ignorance, illiteracy, poverty and poor access to ophthalmic services may be the reasons for this, especially in a suburban environment such as Irrua with its surrounding rural communities.

Juvenile glaucoma was more common in male subjects (male to female ratio was 1.6:1). This is similar to the findings in Benin-City reporting a male to female ratio of 1.5:1.^16^ Early detection of this disorder in children is necessary in order to prevent visual loss. Therefore, a complete ocular examination should be part of preschool medical examinations. Most patients with juvenile glaucoma had myopia. Therefore children with myopia should be routinely screened for this disorder and parents who have glaucoma or myopia should be educated about the need to get their children screened early for glaucoma.

Secondary glaucoma was present in 10 patients (5.6%) with a male to female ratio of 1.5:1. This male preponderance was largely due to the contribution of trauma. Male individuals are usually more frequently involved in traumatic conditions and violence than females. More than half of the secondary glaucomas in this study were due to trauma. The study in Benin-City also reported similar findings.^16^

Twenty-two patients were excluded because of inadequate information mainly on gonioscopy to appropriately classify them. If these subjects were included, the incidence of glaucoma could have been increased to 7.3%. This may also have affected the proportion of patients in various subtypes of glaucoma. It is possible that there were some patients with pseudoexfoliation or pigmentary glaucoma who were not reported in this study. It must be noted however, that pseudoexfoliation and pigmentary glaucoma were not commonly reported in other similar hospital based studies in Nigeria.^16^,^17^

In conclusion, POAG remains the most common type of glaucoma followed by juvenile glaucoma. As much as 51.5% of glaucoma patients were blind at presentation based on visual field criteria. PACG remains relatively rare accounting for only 1.7% of all types of glaucoma. Trauma was the major cause of secondary glaucoma which was the third most common type of glaucoma (5.6%). Congenital glaucoma accounted for only 1.7% of all referred cases. It seems that there is need for prospective community based studies. More awareness campaigns are necessary in order to educate glaucoma patients and communities, and counseling services should be made available to families of sufferers. Educating the general public on the need for early presentation to ophthalmologists and not to other health practitioners may make early diagnosis and management feasible, hence improving the prognosis of the disease.

## Figures and Tables

**Table 1:** Types of glaucoma

	Male	Female	Total	Percentage
Primary open angle glaucoma	81	49	130	73.4
Primary angle closure glaucoma	-	3	3	1.7
Congenital glaucoma	1	2	3	1.7
Juvenile glaucoma	19	12	31	17.5
Secondary glaucomas	6	4	10	5.7
Total	107	70	177	100

**Table 2:** Age and sex distribution of patients with adult and juvenile onset open angle glaucoma

Age (years)	Male	Female	Total	Percentage
≤20	9	4	13	8.1
21–30	10	8	18	11.2
31–40	16	8	24	14.9
41–50	20	12	32	19.8
51–60	22	14	36	22.4
61–70	17	11	28	17.4
71–80	6	4	10	6.2
Total	100	61	161	100

**Table 3:** Stage of glaucoma based on visual field defects

Stage	No.	%
1	38	29.2
2	25	19.2
3	13	10.0
4	4	3.1
5	15	11.5
NA	35	26.9

NA, not applicable.

**Table 4:** Causes of secondary glaucoma

	Male	Female	Total	Percentage
Traumatic hyphema	2	-	2	20
Couching	1	1	2	20
Traumatic iritis	1	1	2	20
Anterior lens dislocation	1	-	1	10
Intumescent cataract	-	1	1	10
Neovascular glaucoma	1	-	1	10
Complicated cataract surgery	-	1	1	10
Total	6	4	10	100
